# Photoreceptor density in relation to axial length and retinal location in human eyes

**DOI:** 10.1038/s41598-022-25460-3

**Published:** 2022-12-09

**Authors:** Songhomitra Panda-Jonas, Jost B. Jonas, Rahul A. Jonas

**Affiliations:** 1 Department of Ophthalmology, University of Heidelberg, 69120 Heidelberg, Germany; 2 Privatpraxis Prof Jonas Und Dr Panda-Jonas, Adenauerplatz 2, 69115 Heidelberg, Germany; 3 Department of Ophthalmology, Medical Faculty Mannheim, Heidelberg University, Mannheim, Germany; 4 Institute of Molecular and Clinical Ophthalmology Basel, Basel, Switzerland; 5 Department of Ophthalmology, University Hospital of Cologne, Cologne, Germany

**Keywords:** Visual system, Retinal diseases

## Abstract

The purpose of the study was to examine the density of retinal photoreceptors and retinal pigment epithelium (RPE) cells in relation to myopic axial elongation in human eyes. Using light microscopy, we assessed the density of photoreceptors and RPE cells at the ora serrata, equator, and midperiphery (equator/posterior pole midpoint), and the RPE cell density additionally at the posterior pole, in enucleated human globes. The study included 78 eyes (mean age: 59.2 ± 15.6 years; range: 32–85 years) with a mean axial length of 27.3 ± 3.6 mm (range: 21.5–37.0 mm). Close to the ora serrata, at the equator and midperiphery, photoreceptor and RPE cell density was 246 ± 183, 605 ± 299 and 1089 ± 441 photoreceptors/mm and 56.1 ± 13.7, 45.2 ± 15.1, and 48.8 ± 15.6 RPE cells/mm, respectively. Densities of both cell types in all three regions were positively correlated with each other (all *P* < 0.001) and decreased with longer axial length (all *P* < 0.001) and longer distance between the ora serrata and the posterior pole (all *P* < 0.001), most marked at the midperiphery and least marked close to the ora serrata. The PRE cell density at the posterior pole was not significantly (*P* = 0.35) related to axial length. The photoreceptor density at the ora serrata (beta:− 0.33) and equator (beta: − 0.27) and RPE cell density at the ora serrata (beta: − 0.27) decreased additionally with the presence of glaucoma. The findings suggest that the axial elongation-related decrease in photoreceptor and RPE cell density is most marked at the midperiphery, followed by the equator and finally the ora serrata region. It suggests that the axial elongation-related enlargement of the eye wall predominantly takes place in the retro-equatorial region, followed by the equatorial region.

## Introduction

Axial myopization is associated with an increase in the sagittal diameter of the globe, and to a lesser degree, with an elongation of the coronal diameters^1–3^. The process changes the shape of the eye from a sphere to a prolate ellipsoid^1, 4, 5^. Although, due to geometrical reasons, the axial elongation-related change in the eye shape suggests that the myopic elongation of the eye wall takes place predominantly in the equatorial region, studies have not comprehensively addressed that question yet. Knowledge of which part of the eye wall elongates in the course of axial myopization is important to further elucidate the etiology of the process of axial elongation in general. We used the local density of the photoreceptors and RPE cells, measured in various ocular regions, and their correlations with axial length as surrogate to assess which parts of the ocular wall enlarged most markedly in axially elongated eyes.

## Methods

The histological study consisted of human eyes which had been removed due to diseases like painful end-stage glaucoma and malignant melanomas of the choroid and ciliary body. According to the guidelines laid down in the World Medical Association Declaration of Helsinki, the Medical Ethics Committee II of the Medical Faculty Mannheim of the Heidelberg University approved the study and waived the necessity of an informed written consent signed by the study participants, since the enucleations had been performed up to 50 years before start of the investigation^6–8^. All patients were of European descent. None of the eyes examined in the study were hypotonic or showed signs which could result in shrunken eyeballs.

After surgical removal of the eyes, they had been placed in a fixative solution containing 4% formaldehyde and 1% glutaraldehyde and kept in the solution for one week. Using a millimeter scale, the sagittal, horizontal and vertical diameter of the fixed unopened globes were measured. The sagittal globe diameter (“axial length”) was defined as the distance between the corneal apex and the scleral outer surface at the posterior pole. An 8 mm thick middle part of the globes was prepared. The optic nerve head, the pupil and the macular region were included in that middle part, unless the globes contained a tumor which was included into the anterior–posterior section. Routinely prepared for a light microscopical examination, the middle part was dehydrated in alcohol, imbedded in paraffin and sectioned for a light microscopical examination. The sections were stained by hematoxylin–eosin or by applying the Periodic-Acid-Schiff method. One section (thickness: about 8 µm) running through the central part of the optic nerve head and pupil was used for the further analysis. The histological slides were prepared within two weeks after surgical removal of the eyes. They were kept in an archive, so that they could be retrieved for the purpose of the study. The methodology of the measurements of the globe diameters and the histological processing was mostly unchanged during the period of 50 years during which the histological sections were collected.

**Figure 1:**
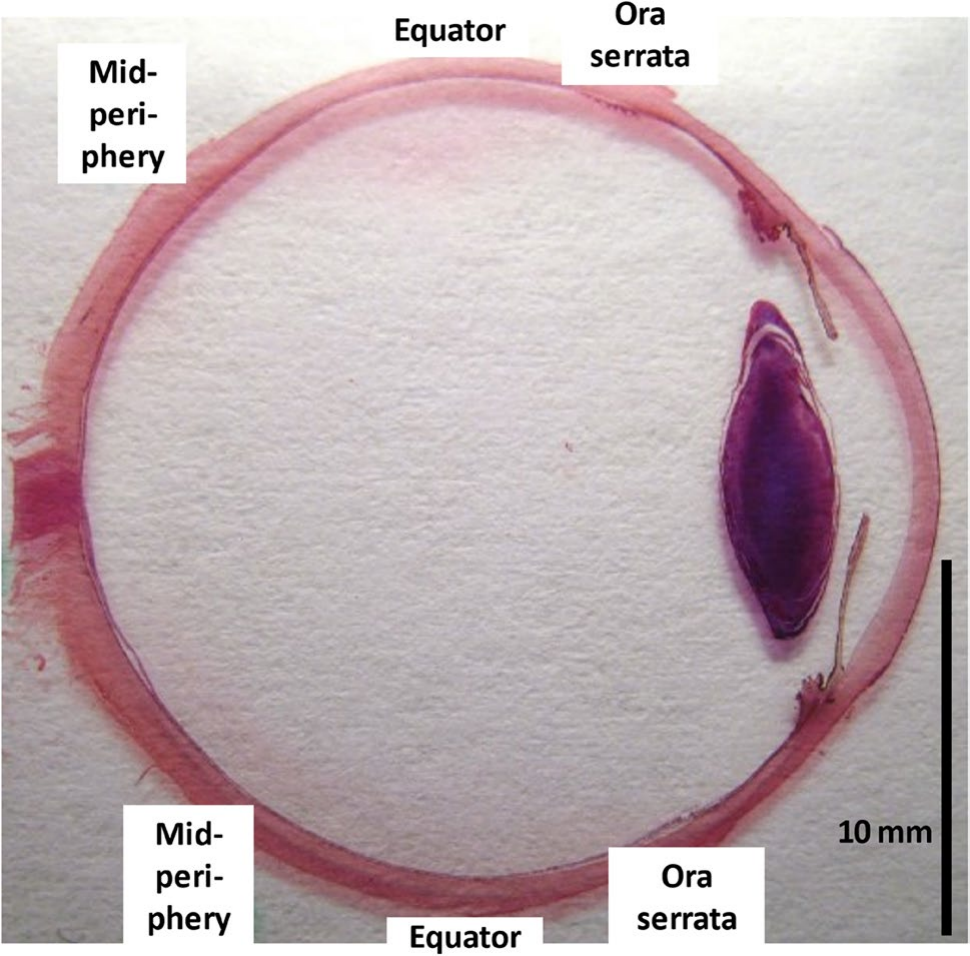
Globe shoring the measurement locations.

Under a conventional light microscope with a magnification of ×250, we counted the number of RPE cells over a length of 480 µm at four measurement points: the posterior pole, the midperiphery (defined as the midpoint between the equator and the posterior pole), the equator, and in the region just posterior to the ora serrata (Fig. 1). For the determination of the length of the measurement region, we used a scale built into the objective of the microscope. Using the same technique, we counted the photoreceptors (defined as nuclei in the outer nuclear layer) over a length of 48 µm at three measurements points: the midperiphery, the equator, and in the region just posterior to the ora serrata. Since the foveal center was included only in a minority of the slides, we did not assess the photoreceptor density at the posterior pole. The assessments of the RPE cell and photoreceptor counts were performed jointly by two examiners (SPJ and JBJ).

While the borderline between moderate myopia and high myopia has not been generally defined, we chose as cut-off value for the definition of high myopia a sagittal globe diameter of ≥ 26.0 mm. Reasons were that an enlargement of Bruch´s membrane opening and the development and enlargement of a myopic parapapillary beta zone usually starts at a myopic refractive error of − 6 to − 8 diopters or an axial length, measured intravitally, of 26.0 to 26.5 mm^9, 10^. Inclusion criterion for the study was the clear detectability of the outer nuclear layer and the RPE cell layer. We assessed the photoreceptor cell count/mm and RPE cell count/mm at both sides of the globe on the histological slide. The region in which a choroidal malignant melanoma was located, was excluded from the examination. The method has already been described in detail previously^6–8, 11^.

Using a statistical software program (SPSS for Windows, version 27.0; IBM-SPSS, Chicago, Illinois, USA), we calculated the mean values, standard deviations and 95% confidence intervals (CI) of the main outcome variables, i.e., the density of the photoreceptors and RPE cells. We tested correlations between these parameters and other histomorphometric parameters such as axial length and the distance between the ora serrata and the posterior pole or the distance between the ora serrata and the scleral spur. The distance between the ora serrata and the posterior pole and the distance between the ora serrata and the scleral spur had been assessed in a previous study, with the retinal length defined as the distance between the ora serrata and the optic disc border, and the ciliary body length defined as the distance between the ora serrata and the scleral spur^12^. In that study, the distance between the ora serrata and the posterior pole elongated by 0.73 mm, and the distance between the ora serrata and the scleral spur increased by 0.16 mm for each mm of axial elongation. The statistical significance of differences between measurement locations was assessed using a within subject, repeated measures ANOVA (analysis of variance). The standardized regression coefficient beta and the non-standardized regression coefficient B and its 95% confidence interval (CI) were determined, and the level of statistical significance was set at < 0.05 (two-sided).

## Results

The study included 78 eyes of 78 patients (30 (38.5%) men; 48 (61.5%) women) with a mean age of 59.2 ± 15.6 years (median: 59 years; range: 32–85 years) and a mean axial length of 27.3 ± 3.6 mm (median: 26.5 mm; range: 21.5–37.0 mm). Out of the 78 eyes, 45 (58%) globes were highly myopic. The study population consisted of 28 (36%) non-highly myopic eyes without glaucoma and with a malignant choroidal melanoma (axial length: 24.0 ± 0.9 mm), 4 (5%) non-highly myopic eyes with glaucoma (axial length: 23.6 ± 1.1 mm), 13 (17%) highly myopic eyes without glaucoma and with a malignant choroidal melanoma (axial length: 28.9 ± 2.3 mm), 26 (33%) highly myopic eyes with glaucoma (axial length: 29.3 ± 2.5 mm), 6 (8%) highly myopic eyes with congenital glaucoma (axial length: 33.5 ± 2.3 mm), and one (1%) non-highly myopic eye without glaucoma and without intraocular tumor (axial length: 24.6 mm).

**Figure 2:**
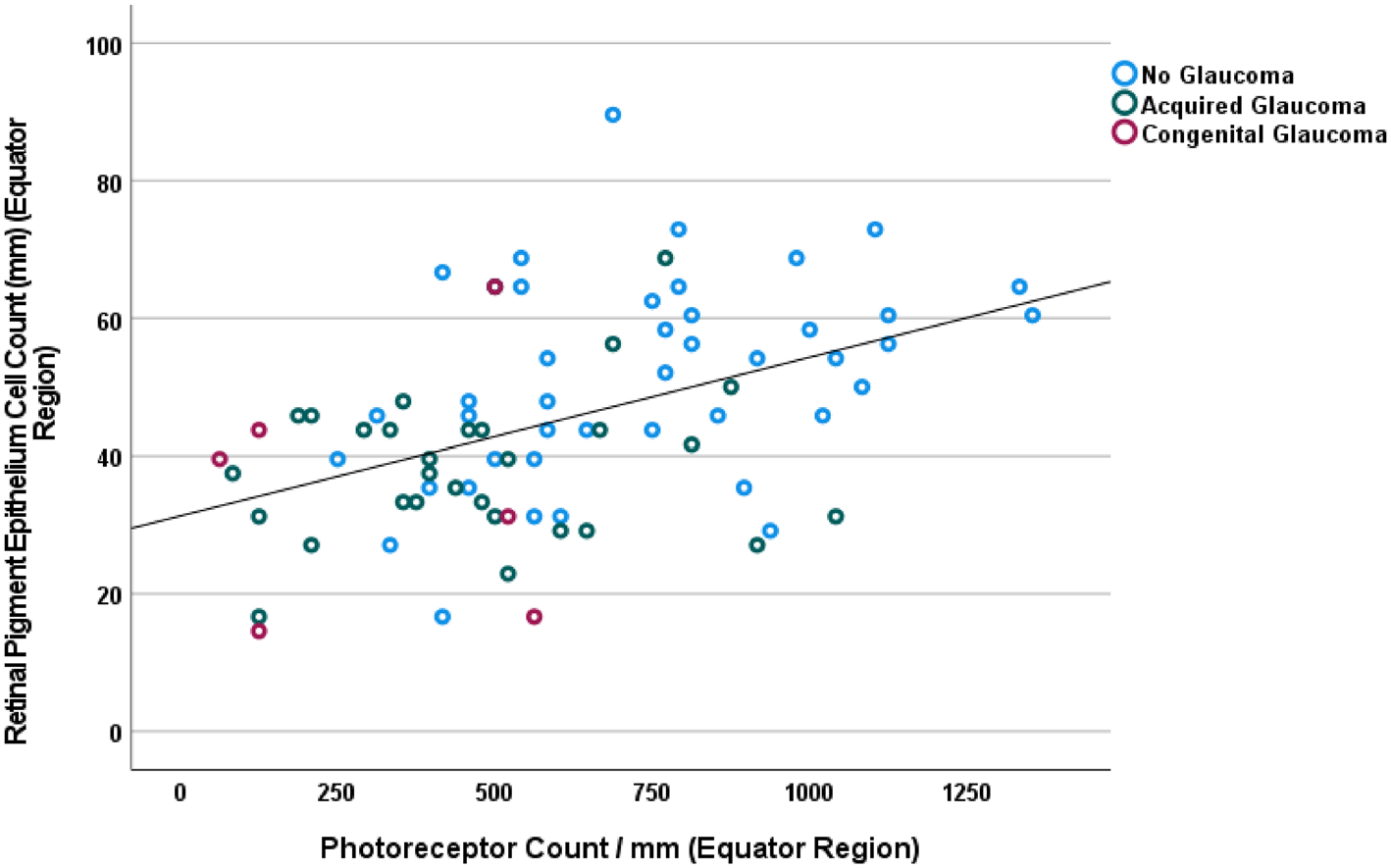
Scattergram showing the distribution of the retinal pigment epithelium cell density at the equator in relationship to the retinal photoreceptor density at the equator.

The photoreceptor count/mm in the midperiphery, at the equator and close to the ora serrata was 1089 ± 441 cells/mm, 605 ± 299 cells/mm, and 246 ± 183 cells/mm, respectively (Table 1). The differences between the measurement locations were significant (*P* < 0.001). The mean RPE cell count/mm in the same regions was 48.8 ± 15.6 cells/mm, 45.2 ± 15.1 cells/mm, and 56.1 ± 13.7 cells/mm, respectively (Table 1). RPE cell count at the posterior pole was 69.1 ± 13.0 cells/mm. The differences between the measurement locations were significant (*P* < 0.001). For each region, the counts of both cell types were positively correlated with each other (Fig. 2). All measured cell count parameters, except for the RPE cell count at the posterior pole, decreased with longer axial length (Table 2).

**Table 1:** Count of the cells in the retinal outer nuclear layer and count of retinal pigment epithelium cells (per mm; mean ± standard deviation; median; range).

Location	Retinal outer nuclear layer cell density (cell count per 1 mm)	Retinal pigment epithelium cell density (cell count per 1 mm)
Close to ora serrata	246 ± 183 (240; 0–667)	56.1 ± 13.7 (54; 23–92)
Equator	605 ± 299 (563; 63–1354)	45.2 ± 15.1 (44; 15–90)
Midperiphery (midpoint between equator and posterior pole)	1089 ± 441 (1083; 292–2417)	48.8 ± 15.6 (48; 17–88)
Posterior pole	–	69.1 ± 13.0 (69; 29–96)

**Table 2:** Associations of the retinal outer nuclear layer (ONL) cell count (per mm) and retinal pigment epithelium (RPE) cell count (per mm), measured in various ocular regions, with axial length.

Parameter	Standardized regression coefficient beta	Intercept on Y-axis	Non-standardized regression coefficient B	95% Confidence intervals of B	*P*-value
ONL cell density, close to ora serrata	− 0.57	1033	− 28.8	− 38.3, − 19.3	< 0.001
ONL cell density, equator	− 0.54	1819	− 44.5	− 60.3, − 28.6	< 0.001
ONL cell density, midpoint equator to posterior pole	− 0.57	2994	− 69.8	− 92.6, − 47.0	< 0.001
RPE cell density, close to ora serrata	− 0.56	114.4	− 2.14	− 2.85, − 1.42	< 0.001
RPE cell density, equator	− 0.53	105.5	− 2.21	− 3.01, − 1.40	< 0.001
RPE cell density, midperiphery (midpoint equator to posterior pole)	− 0.69	130.7	− 2.98	− 3.70, − 2.28	< 0.001
RPE cell density, posterior pole*	0.02	68.4	0.07	− 0.77, 0.02	0.86

*With eyes with congenital glaucoma excluded.

Considering the thickness of the histological section of 8 µm, an RPE cell count of 69.1 cells/mm of the histologic section (measured at the posterior pole) and of 56.1 cells/mm of the histologic section (measured in the midperiphery) converts to approximately 8600 RPE cells/mm^2^ and 7000 RPE cells/mm^2^, respectively, and a photoreceptor count of 1089 cells/mm of the histologic section (measured in the midperiphery) converts to approximately 136,125 cells/mm^2^.

**Figure 3:**
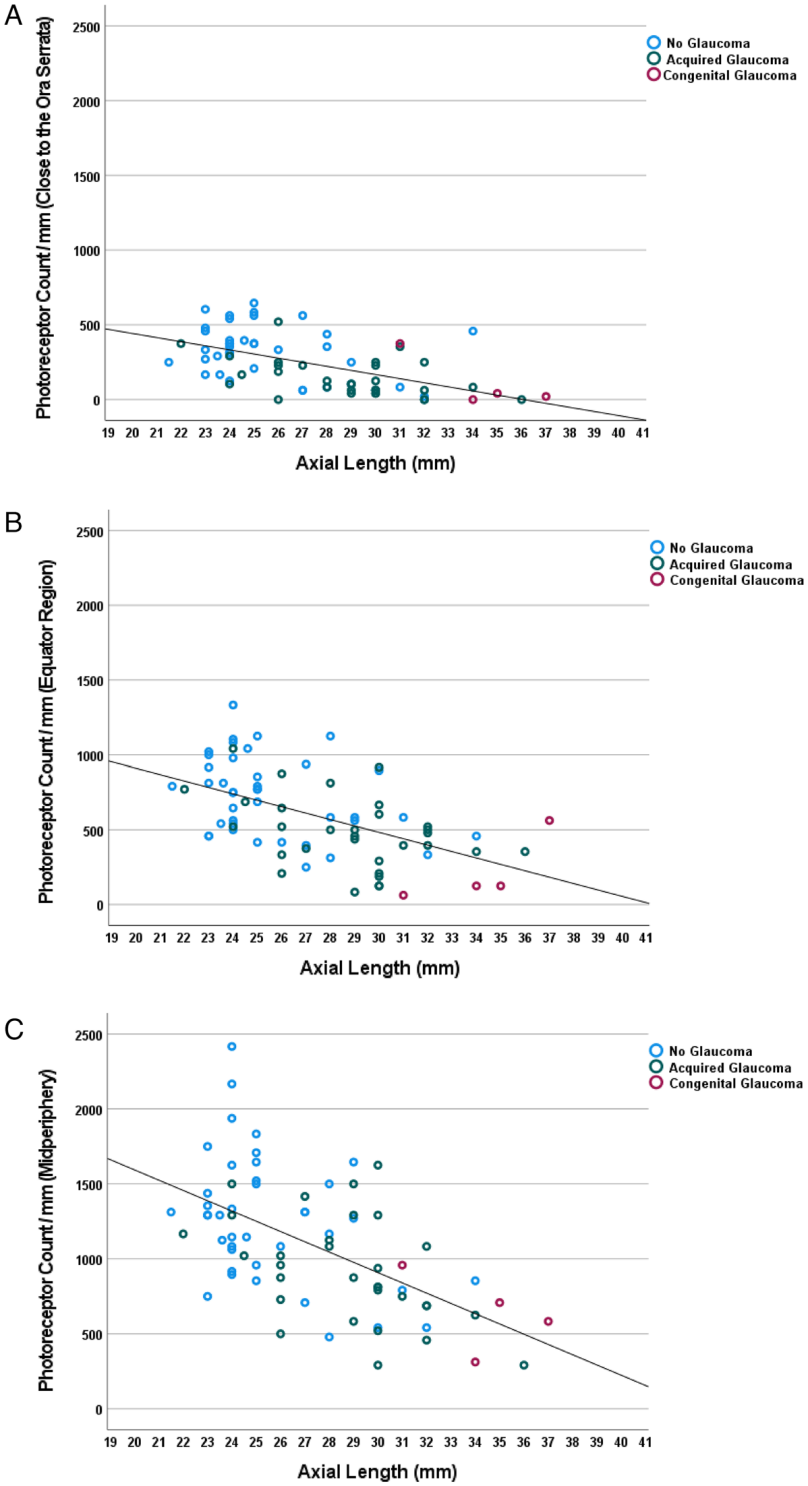
Scattergram showing the distribution of the retinal photoreceptor cell count / mm in relationship to axial length, as measured close to the ora serrata (**a**), at the equator (**b**) and in the fundus midperiphery (midpoint between equator and posterior pole) (**c**). (**a**) Equation of the regression line: Photoreceptor Count/mm = − 28.8 × Axial Length (mm) + 1033. (**b**)Euation of the regression line: Photoreceptor Count/mm = − 44.5 × Axial Length (mm) + 1819. (**c**) Equation of the regression line: Photoreceptor Count/mm = − 69.8 × Axial Length (mm) + 2994.

The axial length-related decrease in photoreceptor cell/mm was most marked in the midperiphery and least marked close to the ora serrata (Tables 2, 3) (Fig. 3a,b,c). The amount of the steepness of the regression line for the associations of axial length with the photoreceptor density was higher in the midperiphery (− 69.8 photoreceptors/mm for each mm of axial length) than in the equatorial region (− 44.5 photoreceptors/mm for each mm of axial length), or in the region close to the ora serrata (− 28.8 photoreceptors cells/mm for each mm of axial length) (Table 2). Similar results were obtained when the correlations between the photoreceptor cell count/mm and the distance between the ora serrata and the posterior pole (“retinal length”) were assessed (Table 3). In a multivariable analysis, the photoreceptor cell count/mm, measured at the equator and in the ora serrata region, additionally decreased with the diagnosis of glaucoma, while it was not significantly correlated with age (all *P* > 0.20) or the diagnosis of a malignant choroidal melanoma (all *P* > 0.20) (Table 4). The results remained mostly unchanged if eyes with congenital glaucoma were excluded.

**Table 3:** Associations of the retinal outer nuclear layer (ONL) cell density and retinal pigment epithelium (RPE) cell density, measured in various ocular regions, with the distance between the ora serrata and the posterior pole.

Parameter	Standardized regression coefficient beta	Intercept on Y-axis	Non-standardized regression coefficient B	95% Confidence intervals of B	*P*-value
ONL cell density, close to ora serrata	− 0.37	671	− 18.3	− 31.6, − 5.0	0.008
ONL cell density, equator	− 0.56	1637	− 43.0	− 61.1, − 24.9	< 0.001
ONL cell density, midpoint equator to posterior pole	− 0.43	2352	− 54.5	− 87.7, − 21.3	0.002
RPE cell density, close to ora serrata	− 0.41	95.9	− 1.68	− 2.77, − 0.60	0.003
RPE cell density, equator	− 0.32	75.7	− 1.31	− 2.41, − 0.21	0.02
RPE cell density, midpoint equator to posterior pole	− 0.58	115.5	− 2.73	− 3.82, − 1.64	< 0.001
RPE cell density, posterior pole	0.69	74.8	− 0.22	− 1.33, 0.88	0.69

**Table 4:** Associations of the photoreceptor density and retinal pigment epithelium (RPE) cell density with axial length and the presence of glaucoma (multivariable analysis).

Parameter	Standardized regression coefficient beta	Non-standardized regression coefficient B	95% Confidence intervals of B	*P*-value
**Photoreceptor density ora serrata region**				
Axial length (mm)	− 0.32	− 16.2	− 27.9, − 4.4	0.008
Presence of glaucoma	− 0.39	− 141	− 226, − 57	0.001
**Photoreceptor density at the equator**				
Axial length (mm)	− 0.35	− 28.5	− 48.7, − 8.4	0.006
Presence of glaucoma	− 0.30	− 178	− 323, − 32	0.02
**Photoreceptor density at the midpoint between equator and posterior pole**				
Axial length (mm)	− 0.42	− 51.3	− 80.7, − 21.9	0.001
Presence of glaucoma	− 0.23	− 206	− 418, 7	0.06
**RPE cell density in the ora serrata region**				
Axial length (mm)	− 0.39	1.47	− 2.39, − 0.56	0.002
Presence of glaucoma	− 0.27	− 7.36	− 14.0, − 0.76	0.03
**RPE cell density at the equator**				
Axial length (mm)	− 0.40	− 1.68	− 2.73, − 0.63	0.002
Presence of glaucoma	− 0.20	− 5.86	− 13.4, 1.7	0.13
**RPE cell density at the midpoint between equator and posterior pole**				
Axial length (mm)	− 0.68	− 2.92	− 3.86, − 1.98	< 0.001
Presence of glaucoma	− 0.02	− 0.70	− 7.48, 6.1	0.84
**RPE cell density at the posterior pole**				
Axial length (mm)	− 0.25	− 0.91	− 1.97, 0.15	0.09
Presence of glaucoma	0.05	1.25	− 6.38, 8.89	0.74

**Figure 4:**
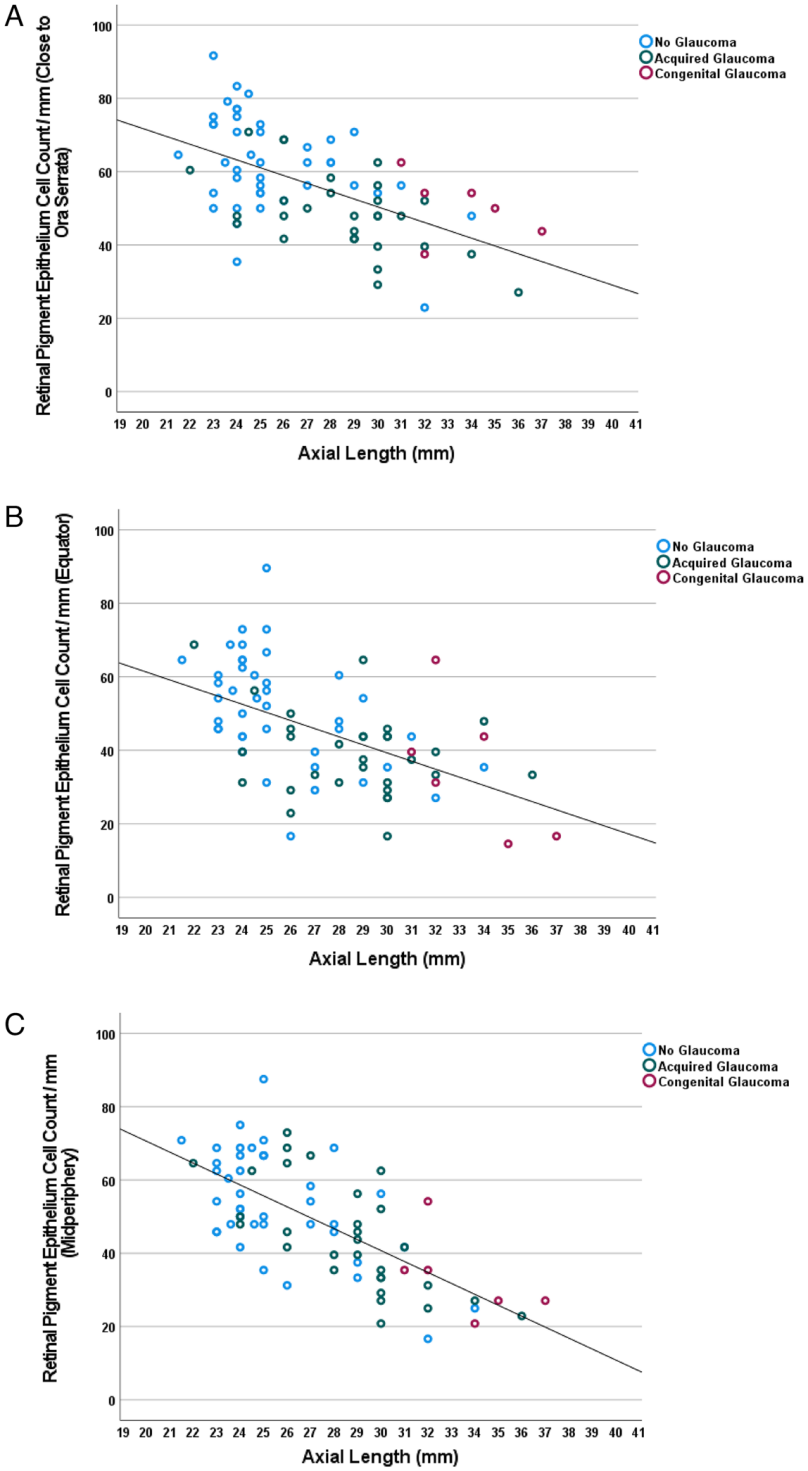

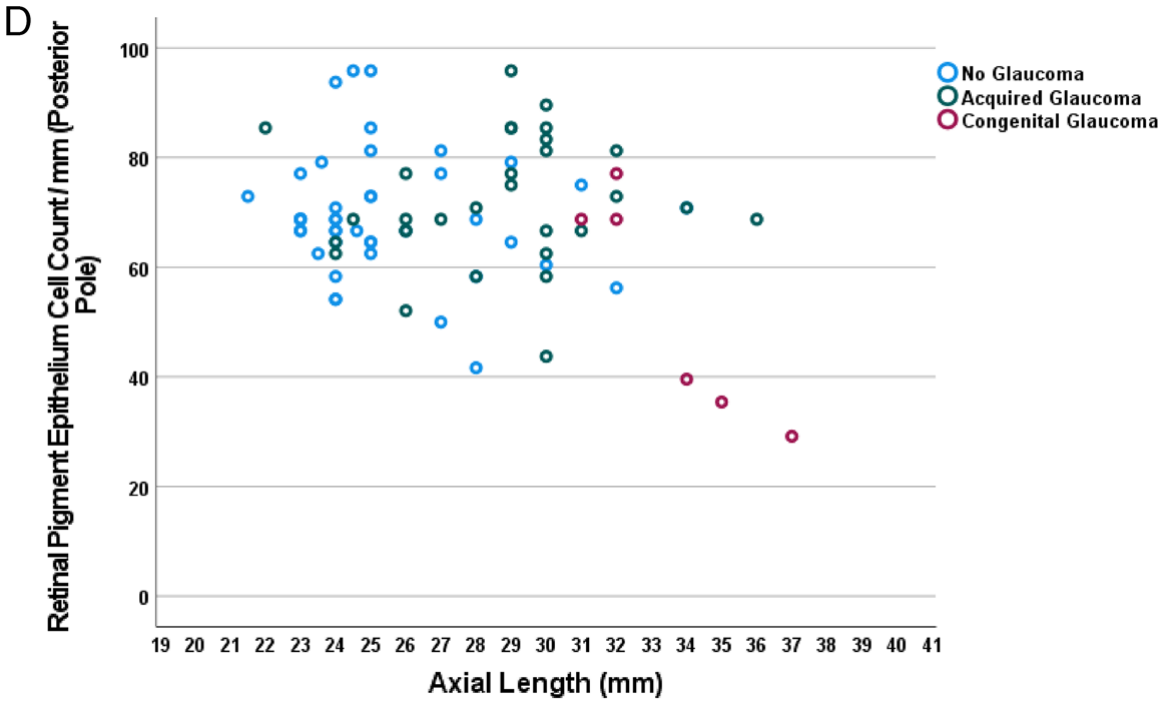
Scattergram showing the distribution of the retinal pigment epithelium cell count / mm in relationship to axial length, as measured close to the ora serrata (**a**), at the equator (**b**) and in the fundus midperiphery (midpoint between equator and posterior pole) (**c**), and at the posterior pole (**d**). (**a**) Equation of the regression line: Retinal Pigment Epithelium Cell Count/mm = − 2.14 × Axial Length (mm) + 114.4. (**b**) Equation of the regression line: Retinal Pigment Epithelium Cell Count/mm =− 2.21 × Axial Length (mm) + 105.5. Figure 4(c) Equation of the regression line: Retinal Pigment Epithelium Cell Count/mm = − 2.98 × Axial Length (mm) + 130.7. (**d**). The association is statistically not significant (*P* = 0.86, with eyes with congenital glaucoma (marked by red circles) excluded).

In a similar manner, the axial length-related decrease in RPE cell count/mm was most marked in the midperiphery (− 2.98 RPE cells/mm for each mm of axial length), followed by the equatorial region (− 2.21 RPE cells/mm for each mm of axial length) and the region close to the ora serrata (− 2.14 RPE cells/mm for each mm of axial length) (Table 2) (Fig. 4). At the posterior pole, the relationship between axial length and RPE cell count/mm was not statistically significant (*P* = 0.052) (Fig. 4d). The absence of a significant correlation between RPE cell count at the posterior pole and axial length became more apparent after excluding the 8 eyes with congenital glaucoma (*P* = 0.86) (Table 2) (Fig. 4d). Within the group of eyes with congenital glaucoma, the RPE cell count/mm, measured at the posterior pole, decreased significantly with longer axial length (beta: − 0.93; B: − 8.46 (95%CI: − 13.3, − 3.6); *P* = 0–008) (Fig. 4d). In a multivariable analysis, the RPE cell count/mm, measured in the ora serrata region, additionally decreased with the diagnosis of glaucoma, while it was not significantly correlated with age (all *P* > 0.20) or the diagnosis of a malignant choroidal melanoma (all *P* > 0.20) (Table 4).

Comparing the cell counts/mm measured on one side of the histological sections with the cell counts/mm on the other side of the histological sections of the globe (e.g., comparing the temporal side with the nasal side) showed no significant differences for any cell count parameter (all *P* > 0.05). The mean difference in cell density between both sides of the histological sections in the midperiphery was 245 ± 231 cells/mm for the photoreceptors and 8.9 ± 6.5 cells/mm for the RPE cells (Table 5). The side differences between both sides of the histological sections in none of the cell density parameters were significantly correlated with axial length (all *P* > 0.05 after Bonferroni correction).

**Table 5:** Side difference in cell counts/mm (mean ± standard deviation; median; range).

Location	Retinal outer nuclear layer cell count/mm	Retinal pigment epithelium cell count/mm
Close to ora serrata	82.9 ± 81.4 (63; 0–354)	11.0 ± 9.7 (8; 0–33)
Equator	186 ± 167 (135; 0–646)	9.7 ± 9.1 (8; 0–42)
Midpoint between equator and posterior pole	245 ± 231 (188; 0–1167)	8.9 ± 6.5 (6; 0–31)

The mean ratio of photoreceptor density to RPE cell density was at the ora serrata, the equator, and in the midperiphery 4.2 ± 3.0, 14.0 ± 7.1, and 23.5 ± 9.4, respectively. The photoreceptor / RPE cell ratio at the equator (*P* = 0.22) and in the midperiphery (*P* = 0.93) was not significantly related to axial length, while the photoreceptor / RPE cell ratio at the ora serrata decreased with longer axial length (beta: − 0.48; *P* < 0.001).

## Discussion

Our study showed that the densities of the photoreceptors and RPE cells in the midperiphery, at the equator and close to the ora serrata were positively correlated with each other and decreased with longer axial length (or alternatively with longer distance between the ora serrata and the posterior pole). The decrease was most marked in the midperiphery and least marked close to the ora serrata. In addition, the RPE cell density at the posterior pole was not related to axial length. The axial length-related decrease in cell density at the equator and in the midperiphery did not differ significantly between the photoreceptors and the RPE cells, so that the ratio of the photoreceptor density to RPE cell density was independent of axial length at those locations. The photoreceptor density at the ora serrata and equator and the RPE cell density at the ora serrata additionally decreased with the presence of glaucoma. Cell densities did not differ significantly between both sides of the eye on the histological section, and differences between both eye sides in photoreceptor and RPE cell density were not significantly related with axial length.

Although it has been known for long that axial myopia is associated with an elongation of the sagittal globe diameter and with an elongation of the ora serrata-optic disc distance, studies measuring the density of the retinal photoreceptors and RPE cells in relationship to axial length have been relatively scarce so far and have been focused mostly on the foveal region^13–19^. It holds true in particular for regional differences in the decrease of the photoreceptor density with longer axial length. The thickness of the whole retina was examined in a previous small-scaled histological study in which the retinal inner nuclear layer and outer nuclear layer, assessed at the equator and in the midperiphery, decreased in thickness with longer axial length^7, 20^. The RPE cell density in relationship to axial length has recently been addressed in another study which as the present investigation found that the RPE cell density decreased with longer axial length in the midperiphery and at the equator, while in that study, the RPE cell density measured in the ora serrata region was not significantly correlated with axial length^8^.

The decrease in the photoreceptor density and RPE cell density being most marked in the fundus midperiphery, followed by the equatorial region and the ora serrata, suggests that the axial elongation-related enlargement of the eye wall predominantly took place in the retro-equatorial region, followed by the equatorial region. These findings and their interpretation fit with the geometry of emmetropic eyes with a spherical shape and with the geometry of myopic eyes with a prolate ellipsoid configuration^1–4^. This change in eye shape may geometrically be explained by a sagittal enlargement of a sphere´s wall in the equatorial region. Interestingly, the photoreceptor and RPE cell density decrease were most marked in the midperiphery, suggesting that the retro-equatorial region is the center of the most marked wall enlargement. If the center of the sagittal wall enlargement were located exactly at the equator, a purely axial elongation would occur. If, however, the center of the wall enlargement is located posterior to the equator, the axial elongation of the globe will be combined with an increase in the horizontal and vertical diameters of the globe. It may explain why axial elongation indeed is associated, to a minor degree, with an enlargement also of the coronal (i.e., the horizontal and vertical) globe diameters^1–4^. It may also explain why axial elongation leads to an enlargement of Bruch´s membrane opening of the optic nerve head^10, 21^. The axial-elongation-related increase in the coronal diameters of the globe may increase the strain within the eye at the posterior pole, leading first to an enlargement of Bruch´s membrane opening, and second, to defects in Bruch´s membrane in the macular region^10, 22^. To assume that the center of the myopic enlargement of the eye wall is located in the retro-equatorial region fits with results obtained in experimental and clinical studies in which the sensory part of the feed-back mechanism regulating the process of axial elongation was discussed to be located in the midperipheral region of the eye^23–25^. The notion of an enlargement of the eye wall, predominantly of Bruch´s membrane, in the retro-equatorial and equatorial region also fits with the clinical observation of a backward shift of Bruch´s membrane opening into the macular direction, explaining an overhanging of Bruch´s membrane into the intrapapillary compartment at the nasal optic disc border, and a compensatory absence of Bruch´s membrane in the temporal parapapillary region, i.e., parapapillary gamma zone^10, 26, 27^. Correspondingly, the optic disc-fovea distance in these eyes is elongated^28^. The hypothesis of a preferential myopic enlargement of the eye wall in the midperipheral region is combined with the notion that a growth primarily of the midperipheral Bruch´s membrane, and not a growth of the midperipheral sclera, may be the driving force elongating the eye^29^. Such a mechanism would push backward the posterior part of Bruch´s membrane, leading to a thinning of the choroid. The axial elongation-related choroidal thinning can geometrically not be explained by a primary backward movement of the posterior sclera. The midperipheral location of the eye wall elongation would also fit with observations made in other studies in which the thickness of the retina and Bruch´s membrane and the choriocapillaris density in the macular region were not correlated with axial length^11, 20, 30–33^. It would also comply with the finding that best corrected visual acuity is mostly independent of axial length if eyes with myopic maculopathy are excluded^34^.

The clinical importance of a decreased photoreceptor density in the retro-equatorial and equatorial region may directly cause a reduction in spatial resolution, and in association with a potentially decreased density of the cells of the inner nuclear layer and retinal ganglion cell layer, an enlargement of receptive fields. Correspondingly, clinical studies have revealed that, at peripheral retinal loci, resolution acuity declined linearly with the magnitude of myopic refractive error^35^. Eyes with a myopic refractive error of − 15 diopters, as compared to emmetropic eyes, had twice as much spacing between retinal receptive units and thus 50% of the peripheral resolution acuity^35^.

The RPE cell density at the posterior pole was not significantly correlated with axial length (Fig. 4d). This finding neither supports nor contradicts the model of retinal stretching in the midperiphery. It does however contradict the model of a uniform global myopic expansion of the eye.

The correlation between a decreased photoreceptor density and glaucoma after adjusting for axial length suggests glaucomatous changes in the deep retinal layers. It agrees with a previous histological study on a different group of eyes, and with previous clinical investigations^36–41^. In experimental studies on rats with acute ocular hypertension, the outer retinal layers thinned after 30 days of elevated IOP, in association with a reduced a-wave and b-wave in the electroretinogram (ERG), and a reduction in the density of cone-related bipolar cells and cones^39–43^. Zhou and associates reported that ocular hypertension led with longer time to a progressive apoptosis first of the retinal ganglion cell layer, followed by apoptosis of amacrine cells in the inner nuclear layer and eventually of cone photoreceptors in the outer nuclear layer^42^. Additionally, presynaptic and postsynaptic vesicle proteins were downregulated. In DBA/2 J mice with essential iris atrophy, pigment dispersion, and glaucomatous optic nerve damage, the eyes showed with increasing age a loss of photoreceptors, in particular of cones, in addition to a loss of horizontal and ON-bipolar cell processes associated with a reduction in synaptic contacts^43^. Other studies on human autopsy eyes of patients with open-angle glaucoma or on monkeys with experimental glaucoma did not confirm glaucoma-related changes in the outer retinal layers^44, 45^. Reasons for the discrepancies between the studies may be the differences in the study period in that in experimental glaucoma in monkeys a potential trans-synaptic degeneration may take longer time as the study lasted and differences in the type of glaucoma.

If the cell density measurements, obtained in our study as cell count per length unit of the histological section, were transformed into cell number per mm^2^, the figure of a photoreceptor density of approximately 136,125 cells/mm^2^ in the fundus midperiphery (calculated from the count of 1089 cells/mm of the histologic section) was similar to the figures found by Curcio and colleagues in whole mounted human retinas^46^. Roughly similar values were obtained also in other studies^47^. The measured count of RPE cells per length unit of the histological slide of 69.1 cells/mm at the posterior pole and of 56.1 cells/mm in the midperiphery translates to approximately 8600 RPE cells/mm^2^ and 7000 RPE cells/mm^2^, respectively. These figures are higher than those reported in studies using human flat mounted RPE tissue, such as an RPE density of 4220 cells/mm^2^ in the fovea 3002 RPE cells/mm^2^ in the fundus midperiphery^48^. In a study on non-human primates, the mean RPE cell density was more than 7000 cells/mm^2^ at the foveal center, and about 4000 RPE cells/mm^2^ in the midperiphery^49^.

The finding of an assumed myopic ocular wall enlargement in the retro-equatorial and equatorial region may have implications not only for a reduced density of photoreceptors and RPE cells in those areas, but it may also indicate an increased distance of these areas from the optic disc. The structures, connecting the middle and deep layers of the retina with the optic disc, are the inner limiting membrane and the retinal ganglion cell axons or retinal nerve fibers. Any elongation of the distance between the retro-equatorial or equatorial region and the optic nerve head may thus lead to an increased strain in the inner limiting membrane and the retinal nerve fibers. An axial-elongation-related increased strain within the retinal nerve fibers may be the reason for the association between a non-glaucomatous optic nerve damage and high myopia, as found in the recent Ural Eye and Medical Study^50^. It has also been discussed that an axial—elongation-related increased strain in the inner limiting membrane may be associated with the development of a myopic maculoschisis^51^.

The limitations of our study should be considered: First, there are more accurate methods ways to assess the photoreceptor density and to test the hypothesis that photoreceptor cell density might reflect axial growth patterns in axially elongating eyes. In a recent study, Ortolan and colleagues used flat mounts of RPE cells and showed rings of varying RPE cell size as a function of distance from the macula, consistent with phases of growth, like rings in a tree trunk^52^. The problem is however that flat mounts are possible only on enucleated eyes and if the eyes were primarily prepared for flat mount-based analysis. Eyes enucleated and prepared in a conventional manner for histological examination as the globes examined in the present study are therefore not suitable for a flat mount-based assessment. Eyes enucleated for clinical reasons primarily demand a histological diagnosis which is not easily obtainable in eyes prepared for a flat mount-based analysis. Furthermore, the rate of globe enucleations has markedly decreased in the last decades, and in particular myopic eyes and highly myopic eyes are only rarely available. Another method would be *en-face* imaging via adaptive optics assisted techniques^53^. While the macular region can well be examined by these techniques, it is difficult to obtain images of the photoreceptor layer in the midperiphery and the far periphery of the fundus. The goal of our study was however to assess the photoreceptor density also in these fundus regions. Second, the photoreceptor density could not be examined at the posterior pole, so that only data on the density of RPE cells at the posterior pole were available for the discussion on the location of the most marked eye globe enlargement in myopia. One may have to be cautious with respect to the RPE cell density at the posterior pole, since previous studies suggested that RPE cells may be able to migrate. Del Priore and colleagues observed in a study on human RPE flat mounts that the density of apoptotic human RPE increased significantly with older age, mostly confined to the posterior pole region^54^. Despite the increased apoptotic rate in the macula, the macular RPE cell density was unchanged with age, while the RPE cell density in the periphery decreased. The authors concluded that peripheral RPE cells may migrate centrally. Using polarimetry, Miura and associates detected an RPE migration in 52 of 155 eyes with age-related macular degeneration, especially in eyes with drusenoid pigment epithelial detachment and serous pigment epithelial detachment^55^. In addition, in a study conducted by Coletta and Watson visual acuity, expressed in cycles/deg and adjusted for spectacle magnification, was not significantly associated with refractive error, while visual acuity expressed as retinal spatial frequency units (cycles/mm) significantly decreased with increasing myopic refractive error^56^. The authors concluded that highly myopic eyes have retinal neurons more widely spaced than emmetropic eyes, but the increased axial length enlarges the retinal image sufficiently to compensate for the retinal enlargement. The findings were in agreement with a model of retinal stretching at the posterior pole. Third, changes in the tissue occurring immediately after the enucleation of the eyes and before the globes were completely fixed, and changes due to fixation-related shrinkage could not be avoided and might have influenced the dimensions of the ocular structures in our investigation. In contrast to donor eyes, the globes examined in the present study may however have undergone autolytic changes since they were fixed within few minutes after enucleation. These changes including mechanically induced alterations of the eyes during the preparation of the histological slides might have led to changes independently of the globe diameters. The procedure-related tissue alterations might thus not have markedly influenced the analysis of relationships of the retinal cell density with axial length. Fourth, the eyes included into our study had been enucleated for various clinical reasons, so that the results of the investigation may not directly be transferred to eyes without these disorders. Fifth, serial sections of the globes were not available. Sixth, statistical significance is not synonymous with practical significance, so that the statistically significant associations between axial length and the photoreceptor and RPE cell density have to be assessed for their practical and clinical meaning and importance. The relatively high correlation coefficients suggest that despite all scattering in the relationships and graphs, the lower cell densities were related to a longer axial length. Seventh, since the patients included in our investigation were of European descent, other investigations may examine whether similar results are obtained in eyes of individuals of other ethnicities.

In conclusion, the axial elongation-related decrease in photoreceptor and RPE cell density was most marked in the midperiphery, followed by the equator and finally the ora serrata region. For the RPE cell density, it was lowest at the posterior pole. It suggests that the axial elongation-related enlargement of the eye wall predominantly takes place in the retro-equatorial region, followed by the equatorial region. The reduced spacing of photoreceptors and RPE cells in the midperiphery and periphery of the fundus in myopic eyes may influence the peripheral resolution acuity.

## Data Availability

The datasets used and analyzed during the current study available from the corresponding author on reasonable request.
